# Measuring high-speed train delay severity: Static and dynamic analysis

**DOI:** 10.1371/journal.pone.0301762

**Published:** 2024-04-10

**Authors:** Bing Li, Chao Wen, Shenglan Yang, Mingzhao Ma, Jie Cheng, Wenxin Li

**Affiliations:** 1 Chengdu Vocational& Technical College of Industry, Chengdu, China; 2 Railway Research Center, University of Waterloo, Waterloo, Canada; 3 China Construction Southwest Consultation & Advisory Co., Ltd, China Southwest Architecture, Chengdu, China; 4 Hubei Key Laboratory of Power System Design and Test for Electrical Vehicle, Hubei University of Arts and Science, Xiangyang, China; 5 Transportation department, China international engineering consulting corporation, Beijing, China; Laboratoire Ville Mobilite Transport, FRANCE

## Abstract

This paper focuses on optimizing the management of delayed trains in operational scenarios by scientifically categorizing train delay levels. It employs static and dynamic models grounded in real-world train delay data from high-speed railways. This classification aids dispatchers in swiftly identifying and predicting delay extents, thus enhancing mitigation strategies’ efficiency. Key indicators, encompassing initial delay duration, station impacts, average station delay, delayed trains’ cascading effects, and average delay per affected train, inform the classification. Applying the K-means clustering algorithm to standardized delay indicators yields an optimized categorization of delayed trains into four levels, reflecting varying risk levels. This static classification offers a comprehensive overview of delay dynamics. Furthermore, utilizing Markov chains, the study delves into sequential dynamic analyses, accounting for China’s railway context and specifically addressing fluctuations during the Spring Festival travel rush. This research, combining static and dynamic approaches, provides valuable insights for bolstering railway operational efficiency and resilience amidst diverse delay scenarios.

## 1. Introduction

Efficient and reliable train operations are fundamental for sustainable transportation systems. However, train delays can dramatically impact the performance and satisfaction of both passengers and railway operators [1]. Precise measurement of train delay severity is essential to identify critical areas, devise mitigation strategies, and enhance overall railway performance. The insights provided will aid researchers and railway authorities in developing effective strategies to reduce delays and improve service quality [2, 3].

Understanding and measuring train delay severity is crucial for maintaining operational efficiency in railway systems. By identifying areas with severe delays, railway authorities can implement targeted strategies to minimize disruptions, improve scheduling, and enhance overall service reliability. Train delays can lead to passenger dissatisfaction and decreased ridership. Accurate measurement of delay severity helps in addressing the root causes of delays and devising solutions to improve the passenger experience, ultimately increasing customer satisfaction. In addition, Severe train delays can have safety implications, leading to overcrowding, increased risk of accidents, and potential security concerns. Proper measurement of delay severity enables early intervention and ensures a safer and more secure transportation environment. Efficient resource allocation is essential for maintaining optimal railway operations. By analyzing delay severity, authorities can allocate resources more effectively to address critical areas and mitigate the impact of severe delays. Studying train delay severity aids in identifying areas with frequent or prolonged disruptions. This knowledge can guide infrastructure planning and maintenance efforts to address vulnerabilities and enhance system resilience. Understanding delay severity trends helps in implementing predictive maintenance strategies, minimizing downtime, and reducing the occurrence of severe delays due to equipment failures. Dynamic measurement of train delay severity provides real-time insights into current disruptions. These real-time data allow railway operators to make informed decisions and adjust operations promptly. In summary, the study of train delay severity measurement, both in static and dynamic ways, carries immense significance for the efficient operation, safety, and customer satisfaction of railway systems. Accurate and timely measurement of delay severity facilitates informed decision-making, resource allocation, infrastructure planning, and emergency response. Additionally, it contributes to the overall optimization of railway services, network resilience, and the improvement of passenger experiences.

This paper investigates the indicators used to gauge the severity of train delays in high-speed railways. A train delay classification model is developed employing the K-means clustering method. By identifying the optimal number of clusters, the model effectively visualizes the delay levels in high-speed railway trains and establishes the relevant classification rules. Additionally, a dynamic analysis model is proposed to assess the severity of train delays in high-speed railways, utilizing a Markov Chain approach to examine the delays from the viewpoints of train stations and trains. The combination of the K-means clustering method with a Markov Chain model in the paper enables a synergistic analysis of train delay severity, where K-means provides insights into static delay patterns and severity levels, while the Markov model offers a dynamic perspective on delay transitions and probabilistic forecasting. This integrated approach enhances the understanding of delay dynamics in high-speed railways and supports informed decision-making for efficient operation and service improvement.

## 2. Literature review

### (1) Factors influencing train delays

Train delays result from a complex interplay of internal and external variables. In an insightful study [4], risk factors affecting disruptions in Shanghai’s metro operation were explored, emphasizing the role of internal factors like equipment failures and operational issues in exacerbating delay severity. Similarly, in the context of benchmarking and evaluating railway operations performance, another study [5] delved into internal elements such as infrastructure conditions and station facilities, shedding light on their substantial impact on railway efficiency. Meanwhile, a notable contribution came from Ref. [6], which introduced a big data analysis approach to assess rail failure risk, with a particular focus on equipment-related factors affecting the reliability of the railway system. Ref. [7], on the other hand, employed predictive models based on high-speed train operation records to delve into the influence of primary delays, with a specific concentration on internal factors that contribute to delay severity.

Furthermore, external factors, especially weather-related issues, have been thoroughly explored in various studies. For example, a study in China [8] concentrated on predicting weather-induced delays in high-speed rail and aviation, underscoring the significance of external factors such as weather conditions in disruption management. Additionally, the impact of weather conditions on rail delays was meticulously analyzed, particularly in the context of Dublin’s metropolitan rail system, highlighting the pivotal role of weather as an external factor affecting rail operations [9].

These studies contribute significantly to understanding the multifaceted nature of train delays. Moreover, these delays and the disruptions they cause have been thoroughly studied in terms of their cost and potential mitigation strategies. A notable study [10] emphasized the financial consequences of equipment-related disruptions, delving into the economic impact of in-service failures of railroad rolling stock. In addition, challenges in railway disruption management and potential solutions have been explored, encompassing strategies for mitigating various factors contributing to disruptions in railway services based on the factors that lead to train delays [11].

This section explores the internal and external variables that contribute to train delays, such as equipment failures, operational issues, infrastructure conditions, and weather-related disruptions. Understanding these factors is essential for identifying the root causes of delays, which is crucial for accurate measurement of delay severity and effective delay management strategies in railway systems. By considering these influencing factors, the paper can establish a comprehensive framework for analyzing and categorizing delay severity levels based on the identified variables.

### (2) Delay prediction methods

Efforts to predict train delays have witnessed a proliferation of diverse methodologies, each enhancing prediction accuracy and operational efficiency. For instance, Ref. [12] introduced a multi-output deep learning model based on Bayesian optimization for sequential train delay prediction, thereby improving the assessment of delay severity and prediction precision. In a similar vein, deep learning approaches were used to predict departure delays at original train stations, combining route conflicts and rolling stock connections to achieve substantial improvements in prediction accuracy [13]. Machine learning techniques have also gained prominence in predicting train delays, with a study introducing network delay measurement using machine learning, successfully transitioning from controlled laboratory settings to real-world deployment in railway systems, thus highlighting the adaptability and practicality of machine learning techniques [14]. Furthermore, resilience planning took center stage in a study by Ref. [15], which developed a multistage decision optimization approach for train timetable rescheduling under uncertain disruptions in high-speed railway networks, holding promise for enhancing the resilience of railway operations and reducing the severity of delays during unexpected events.

The discussion on various methodologies for predicting train delays, including deep learning models, machine learning techniques, and data-driven approaches, highlights the importance of accurate and timely prediction of delays in railway operations. Effective delay prediction methods are essential for anticipating and mitigating delays, which are key components in measuring delay severity and implementing proactive strategies to minimize disruptions. By leveraging advanced prediction techniques, the paper can enhance the dynamic analysis model for assessing delay severity and improving the overall reliability of high-speed railway systems.

### (3) Severity of delays

Understanding the severity of train delays is critical for effective management and mitigation strategies. Ref. [16] significantly contributed to this understanding by analyzing train delays caused by extreme weather events from 2001 to 2020. Their research shed light on the influence of weather-related external factors on the severity of delays in the rail system. Furthermore, Ref. [17] introduced a Markov chain model for delay distribution in train schedules, assessing the effectiveness of time allowances in managing delays. This study aids in comprehending the potential ripple effect of delays throughout schedules. Additionally, the severity of delays can be quantified through delay indices, providing standardized metrics for evaluating train delays and their impact on rail operations [18]. Operations affected by delays in predictive train timetables were the focus of Ref. [19], aiding in schedule optimization and the mitigation of delay severity. The causes of delays in railway passenger transportation were analyzed in another study, providing insights into the factors affecting passenger train delays and the associated severity of these disruptions [20]. Lastly, a combined simulation-optimization approach for robust timetabling on main railway lines was introduced, optimizing timetables for reliability and thereby mitigating the severity of delays in critical railway corridors [21].

The section on the severity of delays emphasizes the significance of measuring delay severity for effective management and mitigation strategies in railway operations. By quantifying delay severity through delay indices and predictive models, the paper can provide standardized metrics for evaluating train delays and their impact on rail operations, aligning with the objective of the study to assess delay severity in high-speed railways. Analyzing the root causes of delays and understanding their severity levels are essential steps in devising targeted interventions to minimize disruptions and enhance service reliability, as highlighted in the literature review.

### (4) Enhancing railway performance while conserving punctuality

Several studies have contributed to the measurement and management of train delays. For instance, Ref. [22] proposed a stochastic model for reliability analysis of periodic train timetables, aiding in timetable optimization and better management of delays. Moreover, a machine learning approach was adopted to predict near-term train schedule performance and delay using bi-level random forests, showcasing the potential of advanced data-driven methods in delay management [23]. Furthermore, delays for passenger trains on a regional railway line were explored, providing a regional perspective on delay factors and their management [24]. Punctuality development and delay explanation factors on Norwegian railways were analyzed, offering a historical perspective on delay trends and contributing to the understanding of delay management strategies [25]. Focusing on determining operations affected by delay in predictive train timetables, a multistage decision optimization approach for train timetable rescheduling under uncertain disruptions in a high-speed railway network was proposed, which has significant implications for enhancing the resilience of railway operations and reducing the severity of delays during unexpected events [26]. These studies collectively emphasize the importance of robust delay measurement and management strategies, showcasing various approaches, including data-driven methods and optimization techniques, to better understand, predict, and mitigate the impact of train delays on rail networks. Some studies underscore the importance of robust timetabling and optimization strategies in reducing the severity of train delays and enhancing railway performance. For example, the disturbance robustness of railway schedules was evaluated, considering various external factors that can disrupt rail services and emphasizing the need for resilient scheduling strategies [27]. Focusing on enhancing railway performance in Norway, factors influencing punctuality and system reliability in the Norwegian railway network were examined [28]. Resilience-based approaches and disturbance robustness are essential for mitigating the impact of train delays. A quantitative analysis approach for resilience-based urban rail systems was introduced, utilizing a hybrid knowledge-based and data-driven method to assess and enhance system resilience [29].

This section underscores the importance of robust delay measurement and management strategies in enhancing railway performance and conserving punctuality. By focusing on optimizing timetables, enhancing system resilience, and reducing the severity of delays during unexpected events, the paper can contribute to the overall improvement of railway operations and service reliability, aligning with the goals of measuring high-speed train delay severity. The literature review provides a foundation for understanding the challenges in delay management and the potential solutions for enhancing railway performance, which can inform the development of effective delay severity measurement models and strategies in the study.

The measurement of delay severity plays a crucial role in effective delay management within railway systems. Understanding the severity of train delays is essential for identifying critical areas, devising targeted mitigation strategies, and enhancing overall railway performance. By quantifying the severity of delays, railway authorities can prioritize resources, optimize scheduling, and improve service reliability to minimize disruptions and enhance passenger satisfaction.

However, there is often a lack of comprehensive delay severity measurement in railway operations, leading to challenges in identifying and addressing the root causes of delays. Without accurate measurement of delay severity, railway operators may struggle to allocate resources efficiently, resulting in prolonged disruptions, decreased operational efficiency, and potential safety risks. The absence of robust delay severity measurement can also hinder the development of proactive maintenance strategies and the timely response to disruptions, impacting the overall resilience and reliability of railway systems.

To address the lackage of delay severity measurement, it is essential for railway authorities to implement systematic approaches for quantifying and categorizing delay severity levels. By utilizing advanced data analysis techniques, such as clustering models and Markov chains, railway operators can gain insights into the impact of delays on operational efficiency and passenger experience. Incorporating delay severity measurement into decision-making processes enables proactive intervention, resource optimization, and the development of targeted strategies to mitigate the impact of delays on railway operations.

In conclusion, the importance of delay severity measurement cannot be overstated in the context of effective delay management in railway systems. By enhancing the measurement and analysis of delay severity, railway authorities can improve operational efficiency, enhance passenger satisfaction, and ensure the safety and reliability of railway services. Addressing the lackage of delay severity measurement through systematic approaches and advanced data analysis techniques is essential for optimizing railway performance and resilience in the face of diverse delay scenarios.

### 3. Data overview

All operational data of the trains in this study were obtained from the CTC system of the High-Speed Railway Dispatching Center of Guangzhou Railway Group. The data pertains to the operational records of trains on the Wuhan-Guangzhou High-Speed Railway for the period from January to November 2016, comprising a total of 437,689 original records. This dataset covers 14 stations along the Wuhan-Guangzhou line, including Guangzhou North Station, Qingyuan Station, Yingde West Station, Shaoguan Station, Lechang East Station, Chenzhou West Station, Leiyang West Station, Hengyang East Station, Hengshan West Station, Zhuzhou West Station, Changsha South Wuguang Plaza Station, Miluo East Station, Yueyang East Station, and Chibi North Station. There are 13 operational sections, with 70 pairs of high-speed trains operating daily on this line. The shortest headway between stations is 5 minutes, and the shortest headway between operational sections can reach 3 minutes.

Table 1 illustrates an example of the raw data, which includes the date of operation, train number, station, actual arrival time, actual departure time, scheduled arrival time, scheduled departure time (with a time precision of 1 minute), and track occupancy information for each train.

**Table 1:** Example of raw train operational data.

Date	Train No.	Station	Actual Arrival Time	Actual Departure Time	Scheduled Arrival Time	Scheduled Departure Time	Occupied Track
2015/03/24	G1002	Guangzhou North	2015/3/24 7:45	2015/3/24 7:45	2015/3/24 7:47	2015/3/24 7:47	II
2016/2/24	D902	Shaoguan	2016/2/24 21:18	2016/2/24 21:18	2016/2/24 21:11	2016/2/24 21:11	II
2016/01/19	G6020	Qingyuan	2016/1/19 15:14	2016/1/19 15:16	2016/1/19 15:09	2016/1/19 15:11	4
2016/1/15	G1008	Lechang East	2016/1/15 12:04	2016/1/15 12:04	2016/1/15 12:01	2016/1/15 12:01	II

### 4. High-speed train delay classification criteria

The crux of grading the extent of high-speed train delays lies in quantifying the scope and consequences of these delays, and assessing potential safety risks in train operations. To achieve this, it is necessary to establish a comprehensive, scientific, and reasonable train delay classification evaluation index system. This system should adhere to principles of comprehensiveness, relative independence, reasonableness, and ease of acquisition for each indicator. The indicators should objectively and sensitively reflect the actual state of train delays while complementing and coordinating with each other to depict the delay situation from various perspectives.

In previous research, some scholars have qualitatively measured the degree of delays, such as using expert scoring methods for quantification, which is subjective and lacks analysis of actual operational data of trains. Other scholars have only selected a single feature variable, such as the number of delayed trains or the duration of delays, which fails to comprehensively and systematically reflect the delay conditions and evolutionary patterns.

In this section, three dimensions are chosen to form the evaluation index system for delay classification. The first dimension relates to the inherent status of delayed trains, described by the time when the delay first occurs. The second dimension pertains to the impact of delayed trains on stations, specifically including the number of stations affected by delays and the average delay per station. The third dimension concerns the impact on trains, encompassing the number of trains affected by delays and the average delay per delayed train.

1. Initial Delay (*t*_1_): This indicator represents the arrival delay of a train at a station due to external disturbances or interference from other trains. It reflects to some extent the delay situation of the train itself and the impact of disturbances on the normal operation of the train. A longer first delay time may have a greater influence on subsequent stations and trains.
2. Number of Stations Affected by Delays (*s*_1_): By setting the delay threshold to 1 minute, this indicator calculates the total number of stations where the arrival time of the train exceeds the threshold. It can be used to assess the longitudinal propagation of train delays.
3. Average Delay per Station (*t*_2_): This indicator is the ratio of the total delay time of the train at all stations to the number of stations affected by delays. It measures the average impact of delayed trains on each station. A longer average delay indicates a more severe delay event.
4. Number of Trains Affected by Delays (*s*_2_): After a train is affected by external disturbances, it may cause delays for subsequent trains, known as chain delays. In this chapter, the number of trains affected by delays includes both the trains initially affected and the subsequent trains experiencing chain delays. This indicator assesses the lateral propagation of train delays, and a higher value indicates a broader range of train delays.
5. Average Delay per Delayed Train (*t*_3_): This indicator is the ratio of the total delay time of affected trains at stations to the number of delayed trains. A longer average delay per delayed train indicates a more severe level of delays.

According to the original data, the five aforementioned delay classification evaluation indicators are calculated for each train, as shown in Table 2. Due to the different attributes of the selected indicators, there are differences in their units and values, making them unsuitable for direct use in clustering algorithms. Therefore, to ensure the application of subsequent models and algorithms, it is necessary to normalize these indicators before modeling. This aims to eliminate differences between indicators as much as possible while retaining the internal information of the indicators to the maximum extent.

**Table 2:** Example of calculated results for train delay classification indicators.

Date	Train No.	*t*_1_ (min)	*s*_1_	*t*_2_ (min)	*s*_2_	*t*_3_ (min)
2016/01/01	D902	2	13	2.92	4	3
2016/01/01	G1002	2	11	4	3	2.21
2016/01/10	D902	6	11	5.73	4	6.4
2016/01/31	G1008	12	13	13.92	10	6.72

The study found that standardized dimensionless methods resulted in more concentrated feature parameter distributions for the delay classification indicator data. Therefore, in this section, the standardized method is chosen to dimensionless the original data, as shown in Table 3.

**Table 3:** Example of standardized dimensionless train delay classification indicators.

Date	Train No.	*Standard*				
		*t*_1_	*s*_1_	*t*_2_	*s*_2_	*t*_3_
2016/01/01	D902	-0.15206	1.288124	-0.18119	0.236107	0.611879
2016/01/01	G1002	-0.15206	0.771122	-0.076017	0.026739	-0.226271
2016/01/10	D902	0.296538	0.771122	0.092459	0.236107	0.244946
2016/01/31	G1008	0.969435	1.288124	0.890042	1.49231	0.280934

### 5. High-speed train delay clustering classification model

#### 5.1 Selection of clustering method

This method exhibits superior performance for data analysis with fewer requirements for the number of clusters and has found extensive applications in fields such as petrochemicals, biomedicine, environmental science, and transportation research. In this section, the K-means clustering algorithm is chosen to achieve the classification of high-speed train delay levels. The K-means is computationally efficient and can handle large datasets, making it suitable for analyzing the extensive real-world train delay data from high-speed railways used in the paper. The method can quickly cluster the data points into groups based on similarity, facilitating the classification of delayed trains into different severity levels. In the context of the paper, where various key indicators such as initial delay duration, station impacts, average station delay, and cascading effects of delayed trains are considered, the scalability of K-means enables a comprehensive analysis of delay dynamics. The results produced by K-means clustering are easy to interpret, as each cluster represents a distinct group of data points with similar characteristics. This interpretability is crucial for classifying delayed trains into different severity levels based on standardized delay indicators, as done in the paper. Dispatchers can swiftly identify and predict delay extents, enhancing mitigation strategies’ efficiency. K-means is a flexible clustering algorithm that allows for the adjustment of the number of clusters based on the data. In the paper, the optimal number of clusters for categorizing delayed trains into severity levels can be determined using the K-means algorithm, providing a customized and optimized classification approach tailored to the characteristics of the train delay data from high-speed railways. K-means is robust to noise and outliers in the data, ensuring that the clustering results are reliable and stable. In the context of analyzing train delay severity, where the data may contain variations and anomalies, the robustness of K-means helps in generating consistent and meaningful clusters that reflect the varying risk levels of delayed trains accurately. By applying the K-means clustering algorithm to standardized delay indicators, the paper effectively classifies delayed trains into severity levels, offering valuable insights for optimizing delay management strategies in high-speed railways.

The entire implementation process of the algorithm is mainly divided into the following five steps:

Step 1: Input the dataset $D=\{x_{i}|x_{i}=(x_{i1},x_{i2},x_{i3},\ldots ,x_{id}),i=1,2,3,\ldots ,n\}$, where $x_{i}=(x_{i1},x_{i2},x_{i3},\ldots ,x_{id})$ represents a d-dimensional vector.

Step 2: Determine initial centroids. Randomly select K sample points from ***D*** as the initial centroids, denoted as $U=\{u_{j}|u_{j}=(u_{j1},u_{j2},u_{j3},\ldots ,u_{jd}),j=1,2,3,\ldots ,k\}$, where $u_{j}=(u_{j1},u_{j2},u_{j3},\ldots ,u_{jd})$ is also a d-dimensional vector.

Step 3: Establish initial partitioning, $C=\{C_{1},C_{2},C_{3},\ldots ,C_{k}\}$, where *C*_*k*_≠∅.

Step 4: Calculate distances and update centroids. Compute the Euclidean distance between sample point *x*_*i*_ and each centroid *u*_*j*_, $d_{ij}=\sqrt{\sum_{l=1}^{d}(x_{il}-u_{jl}{)}^{2}}$. Assign *x*_*i*_ to the category *λ*_*i*_ corresponding to the smallest *d*_*ij*_ and simultaneously update the cluster partitioning, $C_{\lambda_{i}}=C_{\lambda_{i}}\cup \{x_{i}\}$. Calculate the sample mean within the new cluster and use it as the new centroid, ${u_{j}}^{\prime }=\frac{1}{\left|c_{j}\right|}\sum_{x_{i}\in c_{j}}x_{i}$.

Step 5: Determine if iteration is complete. Compare *u*_*j*_ with *u*_*j*_′ to determine if they are equal. If not, return to Step 4; otherwise, stop the calculation, resulting in the final cluster partitioning *u*_*j*_′.

#### 5.2 Method for optimal cluster number selection

During K-means clustering analysis, it is necessary to predefine a value for K, which directly impacts the subsequent clustering results. Therefore, it’s essential to evaluate and select the optimal K value based on the clustering performance under different K values. As clustering algorithms are a type of unsupervised classification with generally no predefined category labels, external evaluation methods, internal evaluation methods, and relative evaluation methods are commonly employed to scientifically assess their effectiveness.

Given that external information for the classification of high-speed train delay levels is unavailable, this section combines internal evaluation methods (Elbow method) and relative evaluation methods (Silhouette Coefficient, Calinski-Harabaz Index, and Davies-Bouldin Index) to select the relatively optimal number of clusters.

#### (1) Elbow method

The Elbow method is an evaluation method for clustering results based on the Sum of Squared Errors (SSE) within the dataset. It can be used for selecting the optimal number of clusters. SSE changes as K varies. When K is less than the actual number of clusters, SSE decreases rapidly as K increases until reaching the ideal number of clusters. When K exceeds the actual number of clusters, SSE decreases gradually and approaches a flat value as K increases. It’s important to note that there is an extreme case where, when K equals *n*, each data point corresponds to a separate cluster, resulting in the minimum SSE. However, this is not the desired outcome. The relationship between SSE and K resembles the shape of an elbow, hence the name "Elbow Method." The formula for calculating SSE is as follows:

$$SSE={\sum_{i=1}^{K}\sum_{p\in C_{i}}\left|p-\overset{¯}{c_{i}}\right|}^{2}$$ (1)

In Eq (1), *C*_*i*_ represents the *i*^th^ cluster, *p* is a sample point in cluster *C*_*i*_, and $\overset{¯}{c_{i}}$ is the mean of all samples in cluster *C*_*i*_.

#### (2) Silhouette Coefficient (SC)

The SC evaluates the quality of clustering results by comparing the dissimilarity between data within clusters and between clusters. A lower dissimilarity within clusters and higher dissimilarity between clusters indicate better clustering results. The formula for calculating SC is:

$$SC(i)=\frac{b(i)-a(i)}{\max\left\{a(i),b(i)\right\}}$$ (2)

In Eq (2), *a*(*i*) represents the average distance of sample *i* to other samples within the same cluster, and *b*(*i*) is the average distance of sample i to samples in other clusters. The *SC*(*i*) value ranges from -1 to 1. A value closer to 1 indicates tighter clustering, signifying better results. A value closer to -1 indicates poorer clustering, while a value of 0 means that the sample lies on the boundary between two clusters.

#### (3) Calinski-Harabaz Index (CHI)

The CHI measures the compactness of points within clusters by calculating the sum of squared distances between points and cluster centers. It also measures the separation of the dataset by calculating the sum of squared distances between cluster centers and the dataset center. CHI is the ratio of separation to compactness. The formula for CHI is:

$$CHI=\frac{\frac{Tr(S_{B})}{K-1}}{\frac{Tr(S_{W})}{n-K}}$$ (3)

In Eq (3), *n* represents the number of clusters, *K* is the current cluster, *Tr*(*S*_*W*_) is the trace of the within-cluster scatter matrix, and *Tr*(*S*_*B*_) is the trace of the between-cluster scatter matrix.

The formula for calculating *Tr*(*S*_*W*_) is:

$$Tr(S_{W})=\sum_{i=1}^{K}\sum_{j=1}^{n}d(x_{j},v_{i})$$ (4)

The formula for calculating *Tr*(*S*_*B*_) is:

$$Tr(S_{B})=\sum_{i=1}^{K}d(v_{i},\overset{¯}{v})$$ (5)

The larger the calculated CHI value, the better the clustering results.

#### (4) Davies-Bouldin Index (DBI)

DBI is an index that reflects the average similarity between clusters. The smaller the value, the better the clustering results, with the minimum value being 0. The formula for DBI is:

$$DBI(k)=\frac{1}{k}\sum_{i,j=1,i\neq j}^{k}\max\frac{s_{i}+s_{j}}{d_{ij}}$$ (6)

In Eq (6), *s*_*i*_ represents the average distance of all samples in class *i* to the class center, and *d*_*ij*_ represents the distance between the centers of classes *i* and *j*.

### 5.3 High-speed train delay static classification results

#### (1) Optimal cluster number selection results

**Fig 1:**
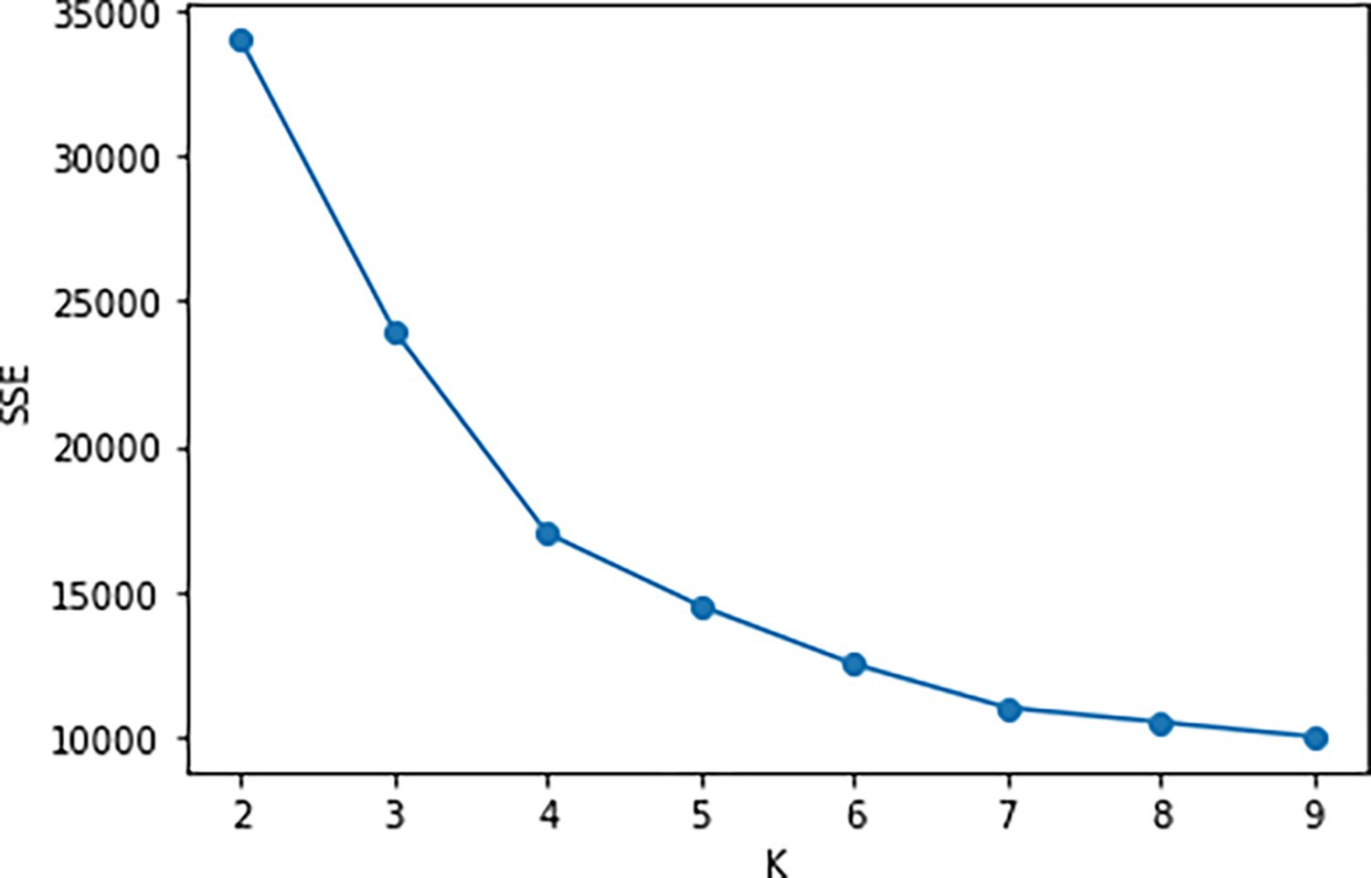
Line graph for determining K value based on elbow method.

Based on Eq (1), the elbow plot as shown in Fig 1 is generated. The results indicate that by using the elbow method, the optimal values for the number of clusters K could be 4, 5, or 6.

Using Eqs (2) to (6), we evaluate the clustering results using the relative evaluation methods SC, CHI, and DBI. This allows us to generate coefficient tables for different values of K, as presented in Table 4.

**Table 4:** Determination of optimal cluster numbers for high-speed train delay data using relative evaluation methods.

Cluster Count	Evaluation Indices		
	SC	CHI	DBI
2	0.707847763*	3810.508942	1.019492622
3	0.419464943	4959.326302	0.943673844
4	0.434634291*	5101.495331*	0.909366194*
5	0.430475804	4659.237552	0.935165392
6	0.304924419	4443.94057	0.968025353
7	0.287533239	4406.690929	1.108612448
8	0.289216731	4262.153808	1.122639372
9	0.292571602	4159.381886	1.088962762
10	0.297905557	4006.005665	1.114953346
Optimal Cluster Count	2, 4	4	4

From Table 4, it can be observed that when evaluating using the Silhouette Coefficient, K = 2 is optimal, while for the other two evaluation indices, K = 4 is optimal. Although K = 2 yields the highest average Silhouette Coefficient, it’s important to note that the SSE is excessively large at this point, as indicated in Fig 1. Therefore, K = 2 is not suitable as the optimal cluster count.

Taking all of the above into consideration, the optimal cluster count for train delays is determined as 4 clusters, meaning that the severity of high-speed train delays will be classified into four levels: A, B, C, and D.

#### (2) High-speed train delay clustering and classification results based on K-means algorithm

**Fig 2:**
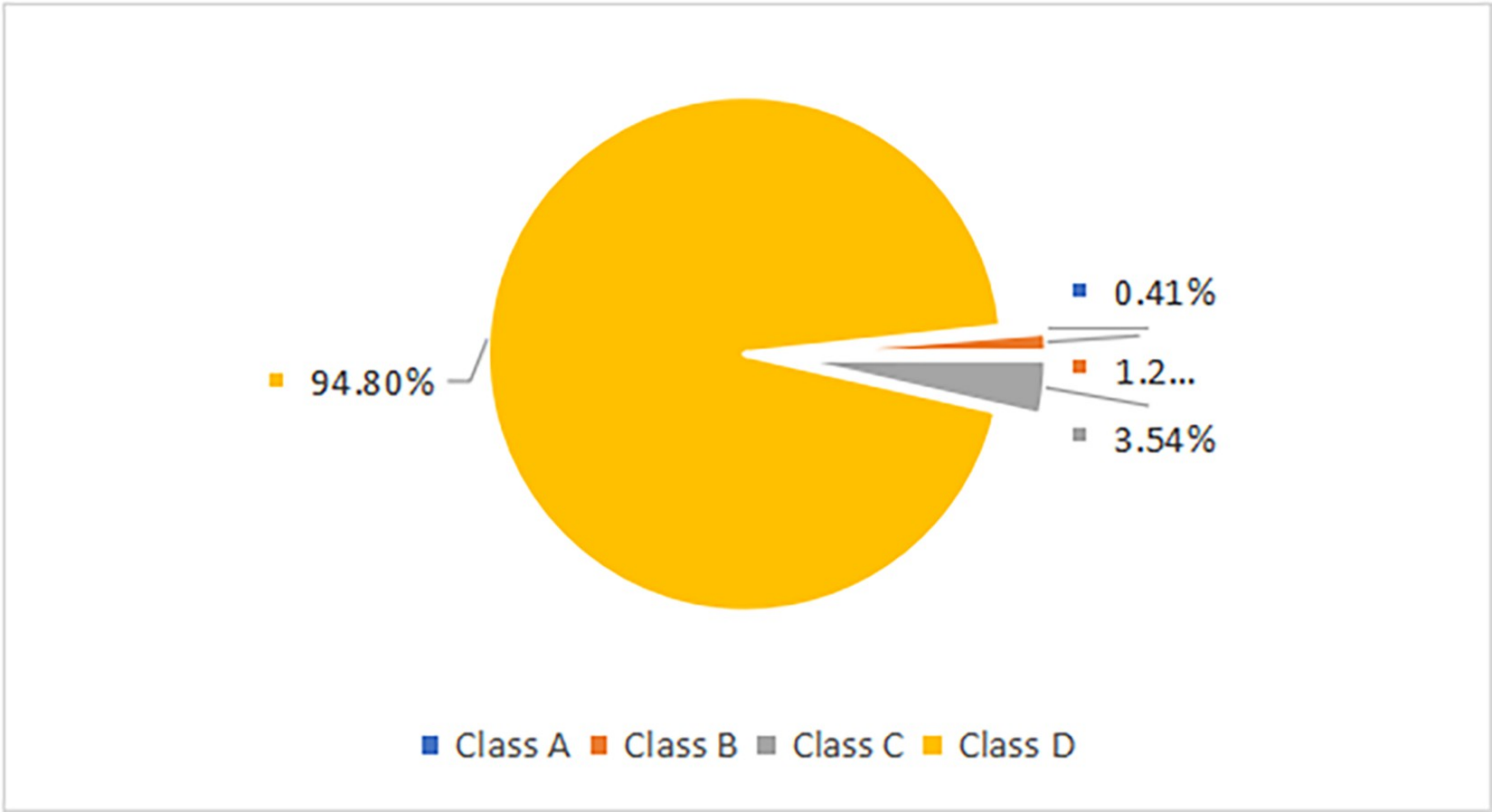
Classification of high-speed train delays.

With K = 4, employing the K-means clustering algorithm, the normalized values of the five delay classification indicators were computed. This clustering was performed on the complete set of high-speed train delay data from the Wuhan-Guangzhou. The total sample count was 10,265. Based on the clustering results, the train delay data was classified into clusters A, B, C, and D, as depicted in Fig 2. Additionally, the centroid of each cluster and the distances between them were computed, as shown in Tables 5 and 6.

**Table 5:** Cluster centers for each train delay classification.

	A	B	C	D
*t*_1_	11.922705	1.829248	0.231335	-0.122764
*s*_1_	0.623407	1.050987	-0.006386	-0.041811
*t*_2_	10.521271	2.936512	0.535621	-0.162053
*s*_2_	1.975849	3.246265	2.235645	-0.159262
*t*_3_	3.909906	5.805223	2.156852	-0.174291

**Table 6:** Distances between cluster centers for each train delay classification.

	A	B	C	D
A	-	12.817	15.507	16.760
B	12.817	-	4.875	5.625
C	15.507	4.875	-	6.489
D	16.760	5.625	6.489	-

#### (3) Visualization of high-speed train delay clustering and classification results

Having selected the scientific train delay classification indicators and performed clustering analysis to obtain reasonable results, further processing may not be necessary from a computational and data mining perspective. However, considering the direction of smart and data-driven high-speed train operations, it’s necessary to visually represent the train delay clustering and classification results to facilitate clear understanding for relevant personnel.

Upon analyzing Table 5, it’s evident that among the five selected train delay classification indicators, the initial delay (*t*_1_), average delay per station (*t*_2_), and average delay per train (*t*_3_) are the three most significant indicators that affect the train delay classification from the most to the least. This analytical result aligns with the actual operational requirements of high-speed train scheduling: In railway operation scheduling, the initial delay is the most intuitive indicator to assess the severity of train delays. A longer initial delay indicates more severe delays. Subsequently, the impact of train delays on stations along the route is a crucial consideration, as overall system safety and stability are paramount in railway scheduling. Finally, the effect of train delays on subsequent trains should be minimized to ensure high-quality passenger services on high-speed railways.

In many fields, there is a need to categorize risks into different levels. For instance, in meteorology, weather warning levels are divided into four categories: very high risk, high risk, moderate risk, and low risk, based on the potential harm, urgency, and development trend of meteorological disasters. Similarly, the response levels for accidents are categorized into four classes: extremely significant, significant, major, and ordinary, following the requirements of the "National Overall Emergency Plan for Public Emergencies (China)" Generally, these levels are represented by colors such as red, orange, yellow, and blue, reflecting the severity from high to low.

**Fig 3:**
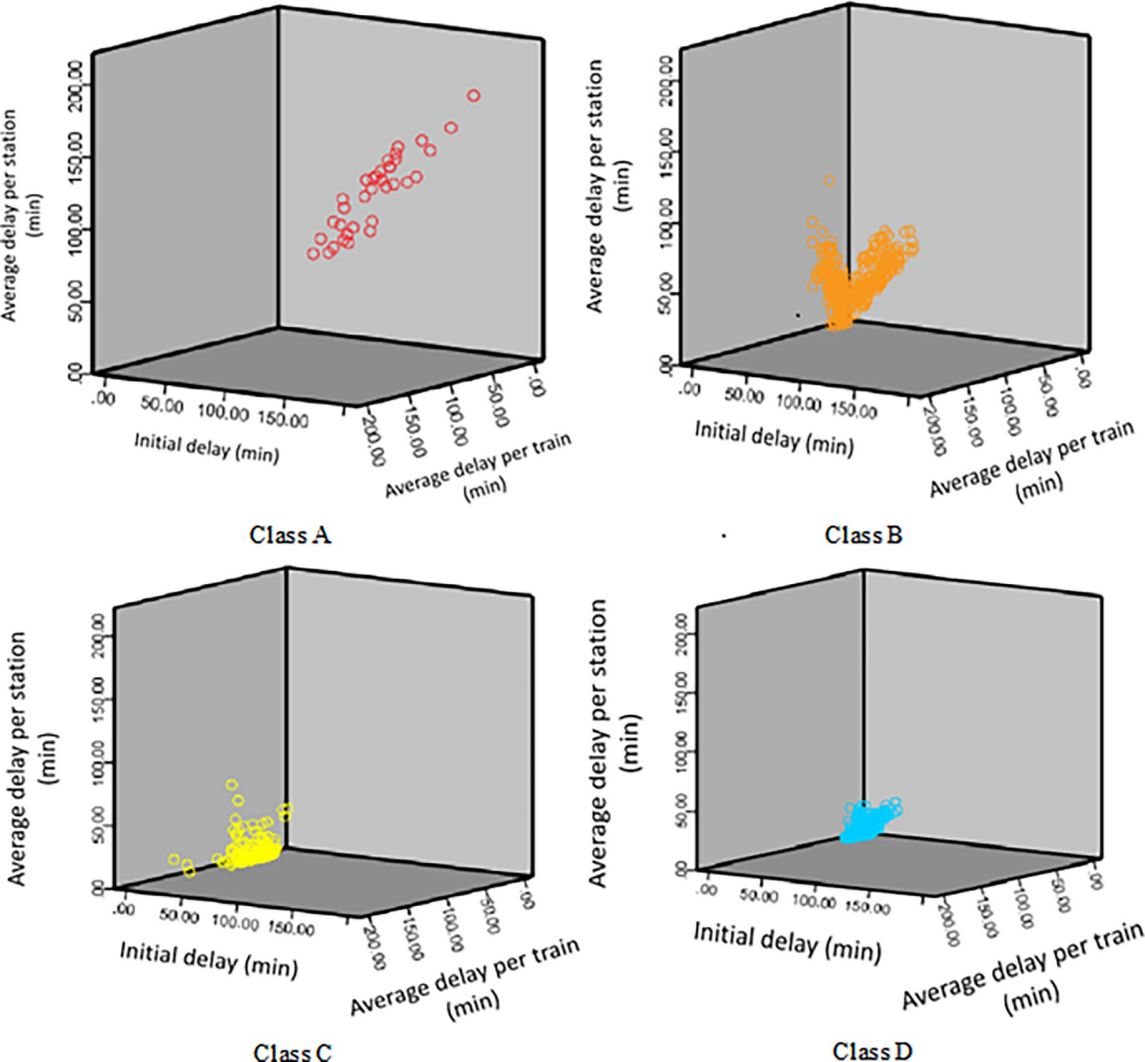
Visualization of classified train delay K-means clustering analysis.

In this section, to ensure accurate judgment by train dispatchers, the visualization of train delay clustering and classification results is based on three dimensions: initial delay, average delay per station, and average delay per train. The results are depicted using colors: red (very high risk), orange (high risk), yellow (medium risk), and blue (low risk), as shown in Fig 3.

It is evident that there are significant differences in the distribution of different categories of delayed trains across the three dimensions. For example, Class A delayed trains exhibit noticeable differences from Classes B, C, and D in terms of average delay per station.

### 5.4 Analysis of high-speed train delay static grading results and grading rules

#### (1) Analysis of high-speed train delay static grading results

From Fig 3, several characteristics of the four classes of delayed trains can be observed:

**A-class Trains:** These trains have the longest first delay time, average delay per station, and average delay per train. This indicates that A-class trains have the most significant impact on the stations along the route and subsequent trains. They are typically the initial delayed trains. In terms of train operation scheduling, the occurrence of delays in A-class trains should be considered as an extremely high-risk situation that can seriously disrupt the transportation organization at various stations.

**B-class Trains:** While these trains have shorter average delays per station compared to A-class trains, they have the longest average delay per train. This suggests that existing transportation organization capacity along the route can alleviate delays for B-class trains, but they can still have a significant impact on subsequent trains. In terms of train operation scheduling, the occurrence of delays in B-class trains should be treated as high-risk situations.

**C-class Trains:** These trains have relatively shorter average delays per station and per train, but their first delay times are generally longer than those of D-class trains. This indicates that the delays in C-class trains are mainly influenced by A-class and B-class trains. In terms of train operation scheduling, the occurrence of delays in C-class trains should be treated as moderate-risk situations.

**D-class Trains:** These trains have the shortest first delay times, average delays per station, and average delays per train. This suggests that the delay severity of D-class trains is the lowest. In terms of train operation scheduling, D-class trains can be considered as low-risk situations.

In the selected dataset, the number of delayed trains for each risk level is as follows: 9731 trains in Level I (Super High Risk), 363 trains in Level II (High Risk), 129 trains in Level III (Medium Risk), and 42 trains in Level IV (Low Risk). It’s noteworthy that the number of trains categorized as high risk might be higher than those in the medium-risk category. Therefore, more effective train organization patterns need to be adopted to minimize the impact of delayed trains on stations and subsequent trains.

#### (2) Grading rules for high-speed train delay static grading

Based on the clustering results of train delay data and considering practical operational circumstances, targeted grading rules for high-speed train delay can be developed. These rules need to provide clear distinctions between different risk levels while also being smooth in their classification.

Considering the three primary indicators that affect train delay grading, a descriptive statistical analysis is conducted on the first delay time (*t*_1_), average delay per station (*t*_2_), and average delay per train (*t*_3_) for different categories of delayed trains. The results are presented in Table 7.

**Table 7:** Descriptive statistical of the three main grading indicators for each category of delayed trains.

Category	Indicator	Minimum	Maximum	Mean	Standard Deviation	Variance	Kurtosis	Skewness
	*t*_1_	1	181	93.40	39.19	1536.17	0.018	-0.132
Class A	*t*_2_	67.18	184.18	102.70	31.98	1022.90	-0.421	0.729
	*t*_3_	5.07	79.99	44.18	21.19	449.22	-0.986	-0.158
	*t*_1_	1	65	17.93	18.54	343.68	0.832	0.732
Class B	*t*_2_	1	66.29	28.21	19.06	363.10	1.151	1.116
	*t*_3_	2.13	184.18	36.20	22.35	499.33	1.545	1.740
	*t*_1_	1	19	6.87	4.05	16.42	0.807	0.868
Class C	*t*_2_	1	33.92	11.33	6.68	44.62	1.976	1.231
	*t*_3_	1	81	10.98	8.75	76.64	2.578	2.257
	*t*_1_	1	5	1.92	1.03	1.07	0.750	1.119
Class D	*t*_2_	1	10.77	2.70	1.51	2.29	2.761	1.436
	*t*_3_	1	20.88	3.35	2.30	5.28	3.464	2.991

By comprehensively analyzing Tables 5–7 and comparing the grading indicators for different categories of delayed trains, the following observations can be made

Class A delayed trains have the highest maximum and mean values for all indicators.

Class A delayed trains exhibit the highest standard deviation and variance, indicating relatively large and dispersed distributions of their grading indicator data.

The kurtosis and skewness values for the grading indicators of A, B, C, and Class D delayed trains show an increasing trend, with Class A trains demonstrating negative kurtosis and skewness values, indicating non-normal distribution.

Based on these findings, a gradual decrease in delay severity from Class A to Class D delayed trains is evident. The corresponding delay levels can be set as follows:

- Level I: Super High Risk
- Level II: High Risk
- Level III: Medium Risk
- Level IV: Low Risk

Taking into account the actual operational data of train delays and the visualized results of clustering analysis, reasonable grading rules for train delays can be established:

Based on the first delay time (*t*_1_):

- Level I:*t*_1_≥ 70
- Level II: 20 ≤ *t*_1_< 70
- Level III: 6 ≤ *t*_1_< 20
- Level IV: 0 < *t*_1_< 6

Based on the average delay per station (*t*_2_):

- Level I: *t*_2_≥ 67
- Level II: 35 ≤ *t*_2_< 67
- Level III: 11 ≤ *t*_2_< 35
- Level IV: 0 < *t*_2_< 11

Based on the average delay per train (*t*_3_):

- Level II: *t*_3_≥ 25
- Level III: 20 ≤ *t*_3_< 25
- Level IV: 0 < *t*_3_< 20

This comprehensive analysis and establishment of grading rules will help improve train scheduling and operation management by providing a systematic approach to categorizing and addressing train delays. By applying these grading rules to all trains’ delay data, a sample of the resulting classification is shown in Table 8.

**Table 8:** Sample of high-speed train delay static grading results of train D902.

*t*_1_	*t*_2_	*t*_3_	Delay Level
2	3	12	IV
3	4	8	IV
4	4	9	IV
4	4	10	IV
3	3	7	IV
4	4	8	IV
6	6	7	III
6	6	7	III
11	11	7	III
5	5	7	IV
5	5	7	IV
7	7	7	IV
11	11	6	III
6	6	7	III
22	22	8	II
7	7	8	III
3	3	8	IV
4	4	9	IV
6	6	9	IV
4	4	9	IV
4	4	8	IV
6	6	9	IV
3	3	9	IV
4	4	8	IV
4	4	8	IV

## 6. Dynamic grading model for high-speed train delays

### 6.1 Markov model for dynamic analysis of high-speed train delays

To understand the variations in delay levels during the train operation and the changes in station delay responses at different time intervals, this section performs a dynamic analysis of train delay levels in both the order of stations and the sequence of time. A Markov chain-based model is established for the dynamic analysis of high-speed train delays as follows:

#### (1) Markov chain state space

Let {***X***(*t*), *t*∈*T*} be a random Markov process, representing the processes of train delay levels during operation and station delay responses adjustment. Let ***E*** be the state space, representing the state set of the propagation chain for train delay levels in station order and the state set of the propagation chain for station delay responses in chronological order.

For $\forall n\in N^{*},\;t_{1}<t_{2}<\cdots <t_{n}<t\in T,\;i_{1}<i_{2}<\cdots <i_{n}<i\in E$, the conditional probability distribution of *X*(*t*) satisfies Eq (7):

$$P\{X(t)=i|X(t_{n})=i_{n},X(t_{n-1})=i_{n-1},\ldots .,X(t_{1})=i_{1}\}=P\{X(t)=i|X(t_{n})=i_{n}\}$$ (7)

In Eq (7), *t*_1_, *t*_2_,….,*t*_*n*−1_ represent "past" states, *t*_*n*_ represents the "current" state, and t represents the "future" state. Through the above process, it is observed that under the condition of knowing states at *t*_1_, *t*_2_,….,*t*_*n*_ and their corresponding states $\{X(t_{1}),X(t_{2}),\ldots .,X(t_{n})\},$ the next state *X*(*t*) only depends on the previous state *X*(*t*_*n*_) and is independent of *t*_1_, *t*_2_,….,*t*_*n*−1_.

Based on the characteristics of the state space, Markov chains can be classified into various types. For example, if the state space is countable, like {1, 2, 3, …, 100}, it is known as a countable state Markov chain. If the state space is finite, it is referred to as a finite state Markov chain.

Among various types of Markov chains, there is a special type that excludes the influence of time and is solely related to the current state on the basis of the Markov chain {*X*(*n*), *n* = 0,1,2,…}. It is referred to as a homogeneous Markov chain, and Eq (8) illustrates this:

$$P(X(k+1)=j|X(k)=i)=p_{ij}$$ (8)

In Eq (8), for all *k*∈*T*, *p*_*ij*_ represents the transition probability from state i to state j.

#### (2) Markov state transition probabilities

Calculating state transition probabilities is a crucial step in applying Markov chains to model and solve practical problems. It represents the probability of a Markov chain transitioning from its current state to another state. Let {*X*(*m*), *m* = 0,1,2,…} be a Markov chain with a state space *E* = {*i*,*j*,…}, and calculate conditional probabilities:

$$p_{ij}^{\left(1\right)}(k)=p\{X(k+1)=j|X(k)=i\}$$ (9)

$$p_{ij}^{\left(n\right)}(k)=p\{X(k+n)=j|X(k)=i\}$$ (10)

Eq (9) represents the one-step transition probability of the Markov chain transitioning from state *i* to state *j* at time *k*. In the model, this corresponds to the process of a delayed train directly arriving at another station and transitioning from one delay level to another, as well as the process of a station adjusting its delay response from one time interval to another in the presence of delayed trains.

Similarly, Eq (10) represents the *n*-step transition probability of the Markov chain transitioning from state *i* to state *j* after n steps at time *k*. In the model, this corresponds to the process of a delayed train arriving at another station through n stations and transitioning from one delay level to another, as well as the process of a station adjusting its delay response from one time interval to another in the presence of delayed trains.

From the formulas of conditional probabilities, it is evident that the n-step transition probability $p_{ij}^{\left(n\right)}(t)$ is not only dependent on states *i* and *j*, but also on the starting point *k*. State transition probabilities exhibit the following properties:

$${p_{ij}}^{\left(n\right)}\geq 0,\forall i,j\in E$$ (11)

$$\sum_{j\in E}p_{ij}^{\left(n\right)}=1,\forall i\in E$$ (12)

The matrix composed of *n*-step transition probabilities is called the n-step transition probability matrix. The one-step transition matrix for a Markov chain can be represented as Eq (13).


$$p^{\left(1\right)}(k)=\left[\begin{array}{ccccc} p_{11}^{\left(1\right)}(k) & p_{12}^{\left(1\right)}(k) & \ldots & p_{1j}^{\left(1\right)}(k) & \ldots \\ p_{21}^{\left(1\right)}(k) & p_{22}^{\left(1\right)}(k) & \ldots & p_{2j}^{\left(1\right)}(k) & \ldots \\ \ldots & \ldots & \ldots & \ldots & \ldots \\ p_{i1}^{\left(1\right)}(k) & p_{i2}^{\left(1\right)}(k) & \ldots & p_{ij}^{\left(1\right)}(k) & \ldots \\ \ldots & \ldots & \ldots & \ldots & \ldots \end{array}\right],E=\{i,j,\ldots \}$$ (13)

It’s worth noting that when the Markov chain is a homogeneous Markov chain, we have *p*^(1)^(*k*) = *p*^(1)^ = *p*. Therefore, the one-step transition matrix of a homogeneous Markov chain can be represented as Eq (14).


$$p=\left[\begin{array}{ccccc} p_{11} & p_{12} & \ldots & p_{1j} & \ldots \\ p_{21} & p_{22} & \ldots & p_{2j} & \ldots \\ \ldots & \ldots & \ldots & \ldots & \ldots \\ p_{i1} & p_{i2} & \ldots & p_{ij} & \ldots \\ \ldots & \ldots & \ldots & \ldots & \ldots \end{array}\right],E=\{i,j,\ldots \}$$ (14)

The n-step transition matrix p^((n)) in this section’s model can be calculated using Eq (15).


$$p^{\left(n\right)}=p^{n}$$ (15)

#### (3) Markov initial distribution

The initial probabilities {*p*_*i*_(0), *i*∈*E*} of the Markov chain at the initial time *t*_0_ are represented as Eq (16):

$$p_{i}(0)=p\{X(0)=i\},i\in E$$ (16)

For ∀*i*∈*E*, the following conditions hold:

$$\begin{array}{c} p_{i}(0)\geq 0\\ \sum_{i\in E}p_{i}(0)=1 \end{array}$$ (17)

After *n* steps of transition, the state distribution *p*_*i*_(*n*) of the Markov chain at time *t*_*n*_ can be calculated as follows:

$$p_{i}(n)=p_{i}(0)p^{\left(n\right)}$$ (18)

And for ∀*i*∈*E*, the following conditions hold:

$$\begin{array}{l} p_{i}(0)\geq 0\\ \sum_{i\in E}p_{i}(n)=1 \end{array}$$ (19)

### 6.2 Dynamic analysis of train delay levels according to station sequence

#### (1) Determination of state set for train delay levels

In this section, the state set of the Markov chain is artificially divided into four train delay states: *R*_1_, *R*_2_, *R*_3_, *R*_4_, which correspond to the four delay levels I, II, III, and IV in terms of train delay levels according to the station sequence.

For this analysis, actual operational data from delayed trains running between Guangzhou North Station and Yingde West Station during the months of January to November 2016 are used. By calculating the state transition probabilities for train delay levels, the aim is to understand the variations in train delay levels. This approach serves the purpose of validating the model’s reliability and avoiding redundant computations. The train delay states are determined according to the train delay classification rules, as illustrated in Table 9.

**Table 9:** Example of calculated train delay status.

Date	Train No.	Departure Station	Delay Level	Delay State
2016/1/1	D902	Guangzhou North	IV	*R*_4_
2016/1/1	D902	Qingyuan	IV	*R*_4_
2016/1/1	D902	Yingde West	IV	*R*_4_

#### (2) Calculation of train delay level transition probabilities

Before calculating the transition probability matrix, it’s necessary to count the frequencies of transitions between different delay level states and obtain an *n*×*n* frequency matrix ***f***, where n is the size of the discrete state set of the Markov chain. The corresponding transition frequency matrix can be obtained from Eq (20).


$$p_{ij}=\frac{f_{ij}}{\sum_{j=0}^{n}f_{ij}},i,j=0,1,2,\ldots ,n$$ (20)

Firstly, based on the actual train performance data (with a sample size of 8146 records), the overall transition probabilities of train delay level states are calculated. Table 10 presents the statistics of transition frequencies between different delay level states for train journeys between Guangzhou North and Qingyuan stations, and between Guangzhou North and Yingde West stations. It is evident that the transition frequencies between delay level states differ within these two intervals.

**Table 10:** Transition frequency of train delay level states between stations.

State Transition	Frequency (Guangzhou North to Qingyuan)	Frequency (Qingyuan to Yingde West)	Frequency (Guangzhou North to Yingde West)
*R*_1_−*R*_1_	42	44	42
*R*_1_−*R*_2_	0	0	0
*R*_1_−*R*_3_	0	0	0
*R*_1_−*R*_4_	0	0	0
*R*_2_−*R*_1_	2	5	7
*R*_2_−*R*_2_	256	339	249
*R*_2_−*R*_3_	53	5	50
*R*_2_−*R*_4_	114	2	119
*R*_3_−*R*_1_	0	0	0
*R*_3_−*R*_2_	55	20	68
*R*_3_−*R*_3_	440	949	420
*R*_3_−*R*_4_	88	327	95
*R*_4_−*R*_1_	0	0	0
*R*_4_−*R*_2_	40	2	44
*R*_4_−*R*_3_	803	53	537
*R*_4_−*R*_4_	6253	6400	6515

This table provides the transition frequencies between different train delay level states for journeys between stations. The frequencies are categorized based on the origin and destination stations. For example, "*R*_1_ to *R*_2_" indicates a transition from delay level *R*_1_ to *R*_2_ for the specified station interval.

Based on the statistical results from Tables 4–10, we can obtain a 4x4 frequency transition matrix, denoted as *f*(1), for the train delay grade states between Guangzhou North Station and Qingyuan Station. In this matrix, each row vector represents the frequencies of transitions from a specific state to other states, including itself. This matrix is then used to calculate the corresponding one-step transition probability matrix, denoted as *p*^(1)^(1), as further described by Eq (9):

$$f(1)=\left[\begin{array}{cccc} 42 & 0 & 0 & 0\\ 2 & 256 & 53 & 114\\ 0 & 55 & 440 & 88\\ 0 & 40 & 803 & 6253 \end{array}\right]$$

$$p^{\left(1\right)}(1)=\left[\begin{array}{cccc} 1.000 & 0.000 & 0.000 & 0.000\\ 0.005 & 0.602 & 0.125 & 0.268\\ 0.000 & 0.094 & 0.755 & 0.151\\ 0.000 & 0.006 & 0.113 & 0.881 \end{array}\right],R=\{R_{1},R_{2},R_{3},R_{4}\}$$

Similarly, the frequency transition matrix *f*(2) and one-step transition probability matrix *p*^(1)^(2) for the train delay grade states between Qingyuan Station and Yingde West Station can be obtained. Additionally, the intermediate frequency transition matrix *f*′(1) and the corresponding one-step transition probability matrix *p*′^(1)^(1) for the transition from Guangzhou North Station to Yingde West Station via Qingyuan can also be derived. These matrices would provide further insights into the transition patterns and probabilities of train delay grades between the specified station pairs.


$$f(2)=\left[\begin{array}{cccc} 44 & 0 & 0 & 0\\ 5 & 339 & 5 & 2\\ 0 & 20 & 949 & 327\\ 0 & 2 & 53 & 6400 \end{array}\right]$$

$$p^{\left(1\right)}(2)=\left[\begin{array}{cccc} 1.000 & 0.000 & 0.000 & 0.000\\ 0.014 & 0.966 & 0.014 & 0.006\\ 0.000 & 0.015 & 0.723 & 0.252\\ 0.000 & 0.001 & 0.008 & 0.991 \end{array}\right]$$

$$f^{\prime }(1)=\left[\begin{array}{cccc} 42 & 0 & 0 & 0\\ 7 & 249 & 50 & 119\\ 0 & 68 & 420 & 95\\ 0 & 44 & 537 & 6515 \end{array}\right]$$

$${p^{\prime }}^{\left(1\right)}(1)=\left[\begin{array}{cccc} 1.000 & 0.000 & 0.000 & 0.000\\ 0.016 & 0.586 & 0.118 & 0.280\\ 0.000 & 0.117 & 0.720 & 0.163\\ 0.000 & 0.006 & 0.076 & 0.918 \end{array}\right]$$

In the same way, one can compute the one-step transition probability matrices for any other pair of stations. The one-step transition probability matrix *p*^(1)^ represents the probability that a train transitions from a certain delay state at station 1 to another delay state after traveling to station 2 with one interval. For instance, in *p*^(1)^(1), the element in the 2^nd^ row and 4^th^ column indicates the probability that a train transitioning from station Guangzhou North to Qingyuan changes its delay state from *R*_2_ to *R*_4_, which is 26.8%.

The Mean Absolute Error (MAE) value (Eq (21)) can be utilized to quantify the differences in the transition probabilities between the three one-step transition probability matrices: *p*^(1)^(1), *p*^(1)^(2), and *p*′^(1)^(1). Calculating *MAE*_*p*_ yields a value of 0.18, indicating that there is relatively little difference in the transition probabilities of delay states between different station pairs.


$$MAE=\frac{1}{16}\sum \max(|p_{ij}^{\left(1\right)}(1)-p_{ij}^{\left(1\right)}\left(2\right)|+|p_{ij}^{\left(1\right)}(2)-p_{ij}^{\prime (1)}(1)+|p_{ij}^{\left(1\right)}(1)-p_{ij}^{\prime (1)}(1)|)|$$ (21)

Hence, the propagation chain of train delay states in sequence can be treated as a homogeneous Markov chain. Further, Eq (15) can be applied to calculate the two-step transition probability matrix. This matrix represents the probability that a train, starting from a certain delay state at station 1, transitions to another delay state after traveling to station 3 with two intervals. Calculating it yields the two-step transition probability matrix from Guangzhou North station to Yingde West station:

$$p^{\left(2\right)}(1)={\left[p^{\left(1\right)}(1)\right]}^{2}=\left[\begin{array}{cccc} 1.000 & 0.000 & 0.000 & 0.000\\ 0.007 & 0.376 & 0.200 & 0.417\\ 0.000 & 0.129 & 0.598 & 0.273\\ 0.000 & 0.019 & 0.186 & 0.795 \end{array}\right],R=\{R_{1},R_{2},R_{3},R_{4}\}$$

The calculation can be continued to derive the three-step transition probability matrix:

$$p^{\left(3\right)}(1)={\left[p^{\left(1\right)}(1)\right]}^{3}=\left[\begin{array}{cccc} 1.000 & 0.000 & 0.000 & 0.000\\ 0.009 & 0.248 & 0.245 & 0.499\\ 0.000 & 0.136 & 0.498 & 0.366\\ 0.000 & 0.033 & 0.233 & 0.734 \end{array}\right],R=\{R_{1},R_{2},R_{3},R_{4}\}$$

Due to China’s unique circumstances, the impact of the return of students and migrant workers to their hometowns results in a significant population movement nationwide within around 40 days centered around the Chinese New Year, known as the Spring Festival travel rush or "Chunyun" period. During this time, there is a substantial increase in national passenger traffic, and the probability of unexpected incidents rises significantly, placing greater pressure on the punctual operation of high-speed trains and the adjustment and recovery of delayed trains. Therefore, in this section, a specific focus is placed on calculating the transition probabilities of train delay levels using actual train performance data during the 2016 Spring Festival travel rush period (from January 24^th^ to March 3^rd^), comprising a dataset of 3433 samples. The aim is to explore differences in the dynamic changes of train delay levels during this period compared to overall patterns.

The transition frequencies between delay level states for trains running from Guangzhou North to Qingyuan stations during the Spring Festival travel rush period are calculated and summarized in Table 11.

**Table 11:** Transition frequencies of train delay level states from Guangzhou North station to Qingyuan station during the Spring Festival travel rush period.

State Transition	Frequency (Guangzhou North to Qingyuan)
*R*_1_−*R*_1_	23
*R*_1_−*R*_2_	0
*R*_1_−*R*_3_	0
*R*_1_−*R*_4_	0
*R*_2_−*R*_1_	0
*R*_2_−*R*_2_	63
*R*_2_−*R*_3_	16
*R*_2_−*R*_4_	42
*R*_3_−*R*_1_	0
*R*_3_−*R*_2_	22
*R*_3_−*R*_3_	209
*R*_3_−*R*_4_	37
*R*_4_−*R*_1_	0
*R*_4_−*R*_2_	15
*R*_4_−*R*_3_	378
*R*_4_−*R*_4_	2628

From Table 11, the 4x4 frequency transition matrix *f*_*s*_(1) of train delay level states from Guangzhou North Station to Qingyuan Station during the Spring Festival travel rush period can be obtained. Based on Eq (9), the corresponding one-step transition probability matrix $p_{s}^{\left(1\right)}(1)$ can be derived. The aforementioned study based on the actual train operation data from January to March 2016 revealed that the propagation chain of train delay levels based on station order can be regarded as a Markov chain. Since this study’s time frame includes data from the Spring Festival travel rush period, the propagation chain of train delay levels during this period can also be considered a homogeneous Markov chain. This enables us to further utilize Eq (15) to compute the two-step and three-step transition probability matrices:

$$f_{s}(1)=\left[\begin{array}{cccc} 23 & 0 & 0 & 0\\ 0 & 63 & 16 & 42\\ 0 & 22 & 209 & 37\\ 0 & 15 & 378 & 2628 \end{array}\right]$$

$$p_{s}^{\left(1\right)}(1)=\left[\begin{array}{cccc} 1.000 & 0.000 & 0.000 & 0.000\\ 0.000 & 0.521 & 0.132 & 0.347\\ 0.000 & 0.082 & 0.780 & 0.138\\ 0.000 & 0.005 & 0.125 & 0.870 \end{array}\right],R=\{R_{1},R_{2},R_{3},R_{4}\}$$

$$p_{s}^{\left(2\right)}(1)=\left[\begin{array}{cccc} 1.000 & 0.000 & 0.000 & 0.000\\ 0.000 & 0.284 & 0.215 & 0.501\\ 0.000 & 0.107 & 0.637 & 0.256\\ 0.000 & 0.017 & 0.207 & 0.776 \end{array}\right],R=\{R_{1},R_{2},R_{3},R_{4}\}$$

$$p_{s}^{\left(3\right)}(1)=\left[\begin{array}{cccc} 1.000 & 0.000 & 0.000 & 0.000\\ 0.000 & 0.168 & 0.268 & 0.564\\ 0.000 & 0.109 & 0.543 & 0.348\\ 0.000 & 0.030 & 0.261 & 0.709 \end{array}\right],R=\{R_{1},R_{2},R_{3},R_{4}\}$$

An overall study of the one-step transition probability matrices *p*^(1)^(1), *p*^(2)^(1), and *p*^(3)^(1) reveals the following:

- Level I delayed trains departing from Guangzhou North Station, even after adjustments over three station intervals, still struggle to reduce their delay levels. This indicates a severe level of delay for Level I trains, requiring more potent measures for rectification.
- Level II delayed trains passing through three station intervals from Guangzhou North Station have their probabilities of transitioning to Level III and Level IV delays increased from 12.5% and 26.8% to 24.5% and 49.9%, respectively. However, it’s important not to disregard that there is still a probability, albeit less than 0.1%, of Level II delayed trains transitioning back to Level I.
- For Level III delayed trains passing through three station intervals, the probability of transitioning to Level IV delays has also significantly increased, rising from 15.1% to 36.6%. However, due to factors like insufficient attention from dispatch personnel to Level III delayed trains, the probability of transitioning back to Level II has increased by 4.2 percentage points.
- Level IV delayed trains passing through three station intervals still have a probability of over 70% to remain at Level IV delays.

A comparison between the one-step transition probability matrices *p*^(1)^(1) and *p*^(2)^(1) for the segments between Guangzhou North Station and Qingyuan Station and between Qingyuan Station and Yingdexi Station reveals:

- Neither segment effectively mitigates Level I delayed trains.
- For Level II delayed trains, departing from Guangzhou North Station, the probability of a delay level reduction after reaching Qingyuan Station is 39.3%. Conversely, for Level II delayed trains departing from Qingyuan Station, the probability of delay reduction after reaching Yingdexi Station is only 2%. This suggests that the capacity between Qingyuan Station and Yingdexi Station might be lower than that between Guangzhou North Station and Qingyuan Station.
- For both segments, there is effective mitigation for Level III and IV delayed trains, with increased probabilities of delay reduction.

An overall study of the one-step transition probability matrices *p*^(1)^(1), *p*^(2)^(1), and *p*^(3)^(1) during the Spring Festival travel rush period shows:

- Level I delayed trains still struggle to reduce their delay levels even after passing through three station intervals.
- For Level II, III, and IV delayed trains, the probabilities of delay reduction increase as they travel through station intervals. For instance, a Level II delayed train departing from Guangzhou North Station and passing through Qingyuan Station, Yingdexi Station, and Shaoguan Station in sequence has a gradually changing probability of 34.7%, 50.1%, and 56.4%, respectively. It’s also important to note that the incremental increase in the probability of delay level reduction diminishes as the train continues its journey, indicating a upper limit on the probability of delay level reduction during travel.

For Level II and III delayed trains, the probability of their delay levels decreasing during the Spring Festival travel rush period is lower than the overall probability of delay level reduction. For instance, during this period, when a Level III delayed train departs from Guangzhou North Station and undergoes a three-station interval journey to arrive at Shaoguan Station, the probability of its delay level decreasing to Level IV is 34.8%. However, when considering the entire dataset, the probability of a train departing from Guangzhou North Station and arriving at Shaoguan Station shifting from Level III to Level IV delay is 36.6%, which is 1.8 percentage points higher than during the Spring Festival travel rush period. Moreover, for Level IV delayed trains, the probability of their delay levels increasing to Level III during the Spring Festival travel rush period is even higher, at 26.1%, which is 2.8 percentage points more than the overall dynamic analysis results.

The calculation of *p*^(*n*)^(1) can be continued to acquire the transition probabilities of train delay levels after passing through *n* stations when departing from Guangzhou North Station. Based on this, the prediction of train delay levels can be achieved. This enables station dispatch personnel to make effective predictions about the delay level upon train arrival at the station, based on its delay level before reaching the station. This proactive approach facilitates the preparation for the arrival of delayed trains and ensures efficient measures for delay mitigation are in place beforehand.

## 7. Conclusions

In conclusion, this paper has investigated the indicators used to gauge the severity of train delays in high-speed railways and has proposed a train delay classification model and a dynamic analysis model to assess the severity of train delays. The train delay classification model employs the K-means clustering method to effectively visualize the delay levels in high-speed railway trains and establish the relevant classification rules. The dynamic analysis model utilizes a Markov Chain approach to examine the delays from the viewpoints of train stations and trains. Overall, this paper highlights the importance of measuring train delay severity and provides valuable insights into the static and dynamic analysis of train delays in high-speed railways. The main contributions of this paper are:

1. **Integration of Static and Dynamic Analysis.** One of the key innovations of the paper is the integration of static and dynamic analysis approaches to measure high-speed train delay severity. By combining these two methodologies, the paper provides a comprehensive understanding of delay dynamics in railway systems, offering insights into both infrastructure-related delays and real-time disruptions.
2. **Application of K-means Clustering Algorithm.** The utilization of the K-means clustering algorithm to categorize delayed trains into four levels based on standardized delay indicators represents an innovative approach to classifying delay severity. This method enables efficient identification and prediction of delay extents, enhancing the effectiveness of mitigation strategies for railway operators.
3. **Markov Chain Approach for Dynamic Analysis.** The paper introduces a dynamic analysis model utilizing a Markov Chain approach to assess delay severity from the perspectives of train stations and trains. This innovative methodology provides real-time insights into current disruptions, enabling prompt decision-making and adjustment of operations to minimize delays and improve railway efficiency.
4. **Focus on Predictive Maintenance Strategies.** By emphasizing predictive maintenance strategies based on trends in delay severity and equipment failures, the paper offers an innovative solution to reduce downtime, enhance system reliability, and improve service quality in high-speed railways. This proactive approach contributes to the optimization of railway operations and the mitigation of severe delays.
5. **Real-world Application and Practical Implications.** The paper’s focus on optimizing the management of delayed trains in operational scenarios using real-world train delay data from high-speed railways demonstrates a practical application of innovative methodologies in railway delay severity measurement. The insights provided in the paper have direct implications for enhancing railway operational efficiency and resilience in the face of diverse delay scenarios.

The innovation in this paper lies in the novel approach of categorizing train delay severity using a combination of static and dynamic models based on real-world data from high-speed railways. By integrating static analysis with the K-means clustering algorithm and dynamic analysis using Markov Chain, the study offers a unique methodology for understanding and managing train delays. This innovative approach provides valuable insights for optimizing railway operations and enhancing resilience in the face of diverse delay scenarios. These innovations contribute to advancing the field of railway management and delay severity analysis, providing valuable insights for railway authorities and researchers in optimizing delay mitigation strategies and improving overall railway performance.

Building on the current method, we plan to explore advanced predictive modeling techniques and incorporate artificial intelligence algorithms to further enhance the accuracy and efficiency of delay severity measurement. By delving into these cutting-edge technologies, we aim to push the boundaries of innovation in railway delay management and contribute to the advancement of the field.
